# Cardiac anomalies in children with congenital duodenal obstruction: a systematic review with meta-analysis

**DOI:** 10.1007/s00383-023-05449-3

**Published:** 2023-03-26

**Authors:** Adinda G. H. Pijpers, Laurens D. Eeftinck Schattenkerk, Ralph de Vries, Chantal J. M. Broers, Bart Straver, Ernest L. W. van Heurn, Gijsbert D. Musters, Ramon R. Gorter, Joep P. M. Derikx

**Affiliations:** 1 Department of Pediatric Surgery, Emma Children’s Hospital Amsterdam UMC, Location University of Amsterdam, Meibergdreef 9, 1005 AZ Amsterdam, The Netherlands; 2 Medical Library, Vrije Universiteit, Amsterdam, The Netherlands; 3 Department of Pediatrics, Emma Children’s Hospital Amsterdam UMC, Location University of Amsterdam, Meibergdreef 9, Amsterdam, The Netherlands; 4 Department of Pediatric Cardiology, Emma Children’s Hospital Amsterdam UMC, Location University of Amsterdam, Meibergdreef 9, Amsterdam, The Netherlands; 5 Amsterdam UMC, Department of Surgery, Location University of Amsterdam, Meibergdreef 9, Amsterdam, The Netherlands

**Keywords:** Duodenal obstruction, Duodenal atresia, Cardiac anomalies, Children, Trisomy 21

## Abstract

**Background:**

Cardiac anomalies occur frequently in patients with congenital duodenal obstruction (DO). However, the exact occurrence and the type of associated anomalies remain unknown. Therefore, the aim of this systematic review is to aggregate the available literatures on cardiac anomalies in patients with DO.

**Methods:**

In July 2022, a search was performed in PubMed and Embase.com. Studies describing cardiac anomalies in patients with congenital DO were considered eligible. Primary outcome was the pooled percentage of cardiac anomalies in patients with DO. Secondary outcomes were the pooled percentages of the types of cardiac anomalies, type of DO, and trisomy 21. A meta-analysis was performed to pool the reported data.

**Results:**

In total, 99 publications met our eligibility data, representing 6725 patients. The pooled percentage of cardiac anomalies was 29% (95% CI 0.26–0.32). The most common cardiac anomalies were persistent foramen ovale 35% (95% CI 0.20–0.54), ventricular septal defect 33% (95% CI 0.24–0.43), and atrial septal defect 33% (95% CI 0.26–0.41). The most prevalent type of obstruction was type 3 (complete atresias), with a pooled percentage of 54% (95% CI 0.48–0.60). The pooled percentage of Trisomy 21 in patients with DO was 28% (95% CI 0.26–0.31).

**Conclusion:**

This review shows cardiac anomalies are found in one-third of the patients with DO regardless of the presence of trisomy 21. Therefore, we recommend that patients with DO should receive preoperative cardiac screening.

**Level of evidence:**

II.

**Supplementary Information:**

The online version contains supplementary material available at 10.1007/s00383-023-05449-3.

## Introduction

Duodenal obstruction (DO) is one of the most common bowel obstructions in neonates and occurs in 1:5000–10,000 live births [1, 2]. After the diagnosis DO is made, the type of obstruction will be discovered during surgery and then be classified following Gray and Skandalakis [3]. Subtypes consist of web/membrane (type 1), atresia (types 2 and 3), annular pancreas, stenosis, and obstruction due to preduodenal portal vein. The type of surgical procedure performed during surgery depends on the subtype and may involve duodeno-duodenostomy, duodeno-jejunostomy, or duodenoplasty [4, 5].

Besides subtypes, associated anomalies can greatly affect perioperative management, especially its associated cardiac anomalies [6]. However, studies describing the association between DO and cardiac anomalies are of small cohort sizes and cannot give clear insights on an association between DO and the specific cardiac anomalies. Increased knowledge on the occurrence of the type of cardiac anomalies associated with DO would highlight the anomalies that shouldn’t be missed throughout the screening process.

In addition to cardiac anomalies, DO is also strongly related to trisomy 21. Trisomy 21 is commonly related with cardiac anomalies. However, whether DO has an increased risk for cardiac anomalies without the presence of trisomy 21 remains unknown.

Therefore, we performed a systematic review with meta-analysis to determine the pooled percentages of cardiac malformation and determine which specific subtype of cardiac anomalies is associated with DO. In addition, we determine the pooled percentages of the different types of obstruction and cardiac anomaly in patients with trisomy 21.

## Methods

A literature review was conducted according to the preferred reporting items for systematic reviews and meta-analysis (PRISMA) guidelines. In accordance with the guidelines, our systematic review protocol was registered in the International prospective register of systematic reviews (PROSPERO) with the number CRD42022302763.

### Literature search

All studies reporting on cardiac anomalies in children with DO were considered eligible for review. The electronic databases were systematically searched by a medical information specialist from inception to July 22, 2022. The following terms were used (including synonyms and closely related words) as index terms or free-text words: “Infants”, “Newborns”, “Duodenal obstruction”, “Duodenal stenosis”, “Duodenal atresia”, and “Annular pancreas”. Age was restricted to one year, no restrictions for languages were applied. Duplicate articles were excluded by a medical information specialist using Endnote X20.0.1 (Clarivate^tm^), following the Amsterdam Efficient Deduplication (AED) method and the Bramer method [7]. Studies were included by two independent authors (LES, AP, and both MD). Any disagreements were resolved by consultation with an expert specialist (JD). The reference lists of the included articles were checked to identify any additional studies of interest. Animal studies, duplicate publications, systematic reviews, congress abstracts, mal-rotation as cause of DO, previous published data, and studies with fewer than 10 patients were excluded. The full search strategies for all databases can be found in the supplementary material.

### Primary and secondary outcomes

The primary endpoint was to determine the pooled percentage of cardiac anomalies in neonates (< 1 year) with DO. Besides this endpoint, a distinction between the subtypes of DO and cardiac anomalies was made. Secondary outcome measures were the pooled percentages of different subtypes of cardiac anomalies consisting of persistent foramen ovale (PFO), persistent ductus arteriosus (PDA), atrioventricular septal defect (AVSD), atrial septal defect (ASD), and ventricular septal defect (VSD). Moreover, the pooled percentages of the different subtypes of DO were calculated. These subtypes were classified as follows: type 1: web/membrane, type 2: fibrous cord, type 3: complete atresia, annular pancreas, stenosis, and preduodenal portal vein. In addition, the pooled percentages per cardiac anomaly in trisomy 21 patients were determined. Finally, a sensitivity analysis was performed to determine the pooled percentages for cardiac anomalies in patients with DO within five different timeframes.

### Data extraction

Two authors (LES and AP) independently extracted the data and evaluated the methodological quality, risk of bias and screened the articles using Rayyan. The full text of the selected articles was obtained for further review. In case of case control studies, the dedicated arm or both arms containing DO patients were used for the analysis. Data on outcome measures were extracted for specific subgroups of patients from the included articles depending on the availability of separate data with regard to specific cardiac malformation and trisomy 21. Disagreements were resolved by discussion between the two reviewers. If no consensus could be reached, a pediatric surgeon was consulted (JD).

### Validity assessment

All included articles were assessed for the methodological quality and risk of bias using the Newcastle–Ottawa quality assessment scale [8].

### Data synthesis

For the cardiac anomalies and each type of obstruction, a weighted average of the logit proportions was determined by the use of the generic inverse variance method. The logit proportions were back-transformed to the summary estimate, and 95% CIs were obtained in a summary proportion representing the pooled percentage of the type of cardiac anomaly, type of obstruction, and trisomy 21. Heterogeneity was assessed using *I*^2^ and *X*^2^ statistics. The random-effects model was used for interpretation. Heterogeneity was deemed significant if the pooled data’s p value was < 0.05 or *X*^2^ statistics were > 75. Heterogeneity was interpreted as small (*I*^2^ < 0.25), medium (*I*^2^ = 0.25–0.50), or strong (*I*^2^ > 0.50), according to Higgins [9].

A sensitivity analysis was performed for five separate time periods: 1956–1979, 1980–1989, 1990–1999, 2000–2009, and 2010–present. These cut-off values were selected in light of the invention and advancement of echocardiography.

## Results

**Fig. 1:**
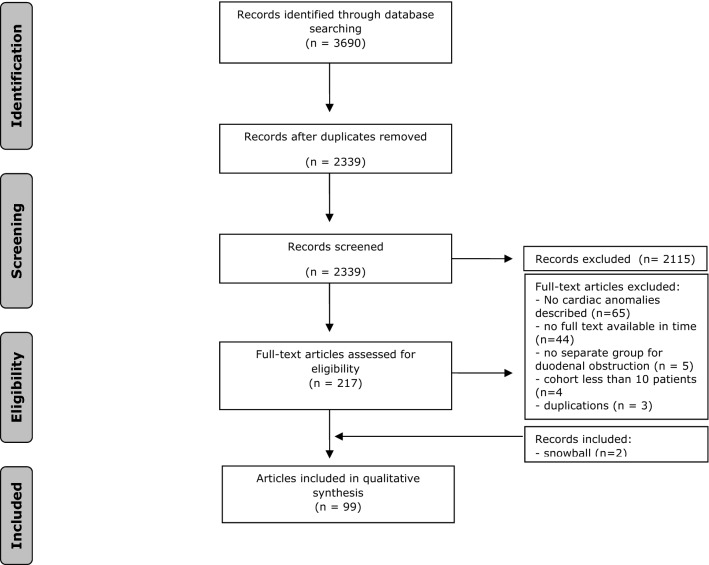
Flowchart of selection

The systematic search resulted in 3683 publications: 1614 in PubMed and 2069 in Embase. After removing duplicates, 2343 articles remained. After screening the title and abstract, 217 were obtained for full text review. In total, 120 articles were excluded because there were no cardiac anomalies described (*n* = 65), no full text available (*n* = 43), no separate data on DOs (*n* = 5), cohort of less than 10 patients (*n* = 4) and duplicates (n = 3). Of the included articles, the reference lists were checked, which resulted in 2 additional eligible studies. In total, 99 articles were included with a total number of 6725 patients and are listed in Table 1 of supplementary materials [1, 6, 10–106]. The flow chart of the search and selection process is presented in Fig. 1.

### Risk of bias

Risk of bias was assessed using the NOS and is shown in Table 2 of supplementary materials. In total, 51 studies were found to have high quality (score 5–9), 47 studies have a high risk of bias (score 2–4), and one study was assessed as very high risk of bias (1).

#### Study characteristics of cardiac anomalies

**Fig. 2:**
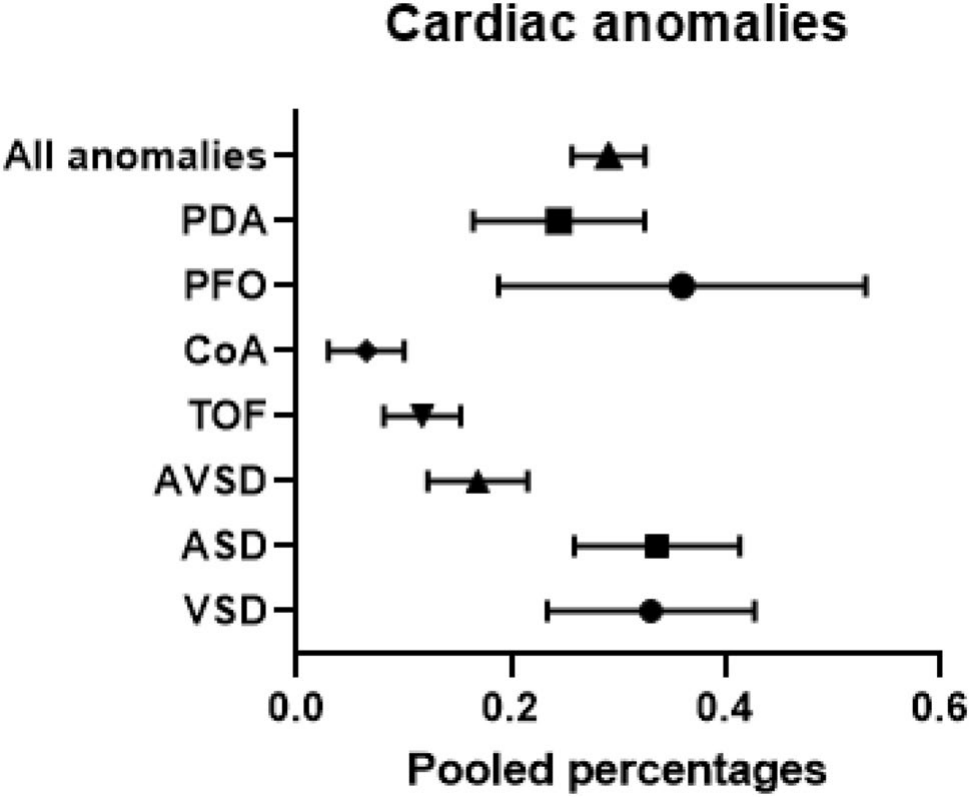
Pooled percentages of cardiac anomalies in patients with DO

The pooled percentage of cardiac anomalies in patients with DO was 29% (95% CI 25.7–32.5; *I*^2^ = 88%; *p* < 0.001) and is shown in Fig. 2. Separate pooled percentages were calculated for the cardiac anomalies in 30 studies, resulting in a total of 671 patients with one or multiple cardiac anomalies [6, 11, 14, 18, 19, 23, 30, 35, 38, 39, 45, 49–52, 57, 58, 61, 64, 67, 68, 83, 85, 91, 96, 97, 100–102]. The most frequent cardiac malformation was PFO and occurred in 35% (95% CI 0.195–0.536; *I*^2^ = 70%; *p* = 0.035) of the patients with DO. This was followed by ASD 33% (95% CI 0.260–0.414; *I*^2^ = 61%; *p* < 0.001) and VSD 33% (95% CI 0.236–0.429; *I*^2^ = 72%; *p* < 0.001). PDA was present in almost a quarter of the studies with a calculated pooled percentage of 24% (95% CI 0.168–0.327; *I*^2^ = 70%; *p* < 0.001). AVSD had a calculated pooled percentage of 17% (95% CI 0.124–0.217; *I*^2^ = 0%; *p* = 0.505) and TOF occurred in 11% (95% CI 0.083–0.154; *I*^2^ = 0%; *p* = 0.11). The least common cardiac anomaly was CoA and occurred in six percent (95% CI 0.033–0.103; *I*^2^ = 0%; *p* = 0.68) of the patients with DO.

### Study characteristics of duodenal obstructions

**Fig. 3:**
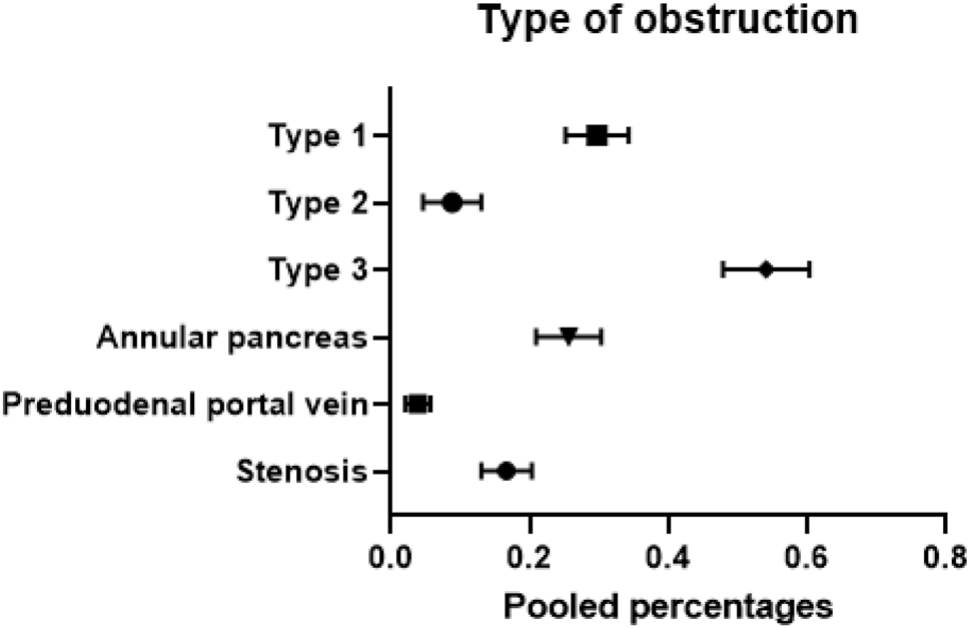
Pooled percentages of type of duodenal obstruction

In total, 70 studies with a total of 4169 patients described the type of DO (Fig. 3) [1, 6, 10–12, 16–19, 21–25, 27–32, 34–52, 54–57, 59, 62–65, 67, 70–75, 81–83, 85, 89, 90, 93–95, 98–100, 102, 105, 106]. Type 3 DO consisting of complete atresia, was the most frequent obstruction and occurred in a pooled percentage of 54% of patients with DO (95% CI 0.478–0.603; *I*^2^ = 88%; *p* < 0.001) of the children. This was followed by the type 1 obstruction, consisting of web/membrane which occurred in 30% of DO patients (95% CI 0.252–0.343; *I*^2^ = 82%; *p* < 0.001) and type 2 in eight percent (95% CI 0.050–0.133; *I*^2^ = 75%; *p* < 0.001) of the patients. Annular pancreas occurred in a pooled percentage of 25% of DO (95% CI 0.210–0.304; *I*^2^ = 91%; *p* < 0.001), and stenosis had a calculated pooled percentage of 16% (95% CI 0.131–0.204; *I*^2^ = 76%; *p* < 0.001). Preduodenal portal vein was the least frequent cause of DO with a pooled percentage of four percent (95% CI 0.022–0.058; *I*^2^ = 0%; *p* = 0.541).

### Study characteristic of cardiac anomalies in Trisomy 21

**Fig. 4:**
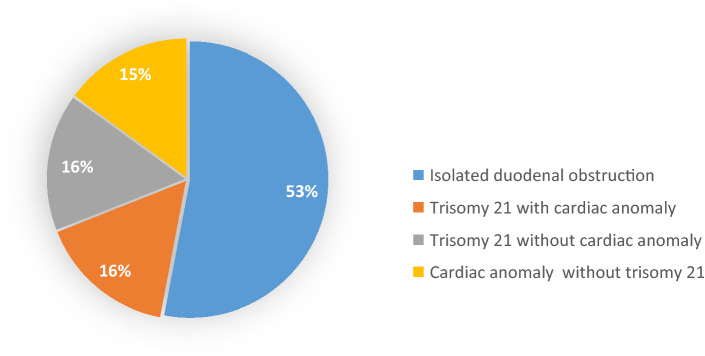
Pooled percentages trisomy 21 and cardiac anomalies

In total, 90 studies described trisomy 21 in 5413 patients with DO [1, 10–12, 14–26, 28–30, 32–67, 69–76, 78–89, 91–95, 97–103, 105, 106]. The pooled percentage of trisomy 21 was 28% (95% CI 0.262–0.308; I^2^ = 66%; p < 0.001) of the patients with DO and is shown in Fig. 4. To determine the pooled percentages for the occurrence of cardiac anomalies with or without the presence of trisomy 21 in patients with DO, we performed a separate calculation. These separate pooled percentages for the presence of trisomy 21 and combination with cardiac anomalies were calculated using 20 studies that described 1552 patients with DO [10, 11, 17, 19, 21, 23, 29, 34, 38, 39, 49, 64, 66, 67, 71, 76, 79, 85, 91, 93, 102]. For this group, the pooled percentage of trisomy 21 in combination with cardiac anomalies was 16% (95% CI 0.123–0.212; *I*^2^ = 77%; *p* < 0.001), and for trisomy 21 without cardiac anomalies, it was also 16% (95% CI 0.131–0.197; *I*^2^ = 57%; *p* < 0.001) of the patients with DO. The pooled percentage for cardiac anomalies without trisomy 21 was 15% (95% CI 0.108–0.196; *I*^2^ = 79%; *p* < 0.001) in patients with DO (see Fig. 2). In these 20 studies, we calculated the pooled proportions for cardiac anomalies in the patients with DO in combination with trisomy 21 existing of 520 patients. This resulted in a pooled percentage of 51% (95% CI 0.413–0.608; *I*^2^ = 70%; *p* < 0.001) for cardiac anomalies in patients with DO and trisomy 21.

**Fig. 5:**
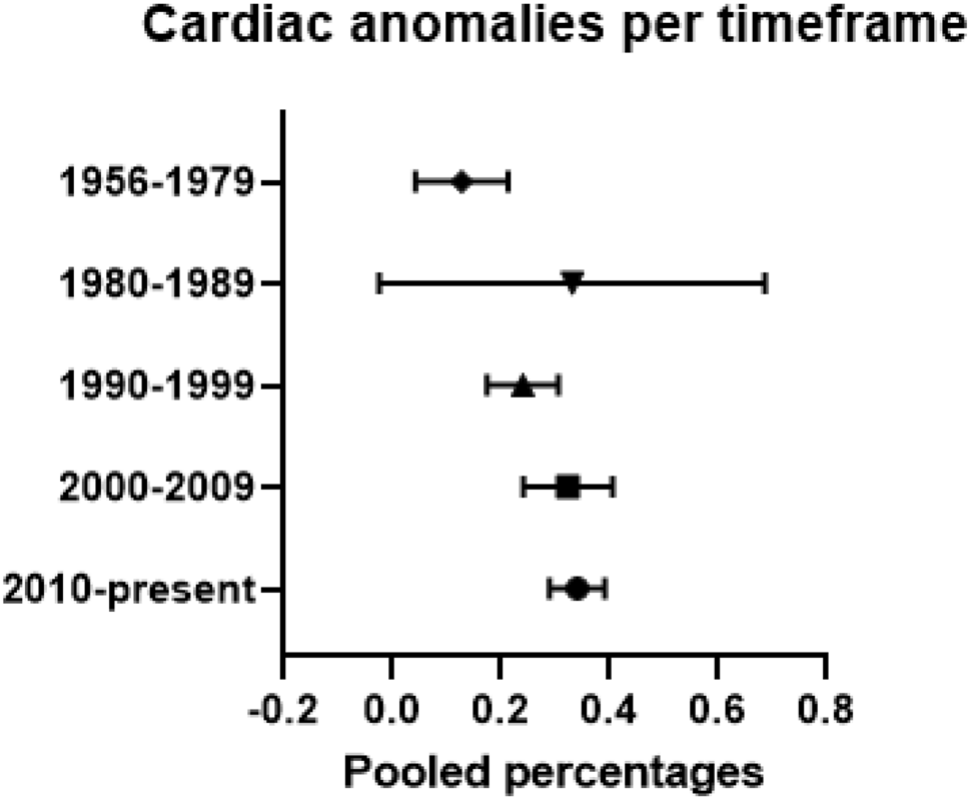
Pooled percentages of cardiac anomalies per time frame

Subtypes of cardiac malformation with or without presence of trisomy 21 were only described in ten studies including 535 patients [11, 19, 38, 39, 49, 64, 67, 85, 91, 102]. Due to the small cohort sizes of subtypes of cardiac anomalies, pooling of the data was only possible for ASD, VSD and PDA. The pooled percentage of VSD in patients with trisomy 21 was nine percent (95% CI 0.063–0.120; *I*^2^ = 12%; *p* = 0.337) and in patients without trisomy 21 seven percent (95% CI 0.048–0.098; *I*^2^ = 0%; *p* = 0.506). This was followed by a pooled percentage of seven percent (95% CI 0.051–0.100; *I*^2^ = 35%; *p* = 0.590) for patients with ASD in combination with trisomy 21, and eight percent (95% CI 0.023–0.253; *I*^2^ = 91%; *p* < 0.001) for those without trisomy 21. The calculated pooled percentages for PDA were six percent (95% CI 0.020–0.154; *I*^2^ = 65%; *p* = 0.034) for patients with trisomy 21 and nine percent (95% CI 0.020–0.301; *I*^2^ = 90%; *p* < 0.001) for patients without trisomy 21 (Fig. 5).

### Study characteristics of cardiac anomalies per timeframe

Pooled percentages of cardiac anomalies were calculated in five different time frames. Between 1956 and 1979, ten studies including 505 patients were described [10, 40, 44, 54, 60, 65, 76, 79, 86, 102]. The calculated pooled percentage was 11 percent (95% CI 0.052–0.220; *I*^2^ = 12%; *p* = 0.323). For the period between 1980 and 1989, nine studies including 524 patients showed a pooled percentage of 23% percent (95% CI 0.034–0.725; *I*^2^ = 95%; *p* = 0.000) [13, 15, 33, 34, 43, 56, 63, 64, 101]. Between 1990 and 1999, 16 studies with a total of 1070 patients were described [11, 17, 30, 32, 38, 39, 41, 51, 61, 66, 70, 75, 80, 89, 96, 99]. The calculated pooled percentage was 23% percent (95% CI 0.177–0.307; *I*^2^ = 70%; *p* = 0.000). The following period between 2000 and 2009 included 17 studies with 970 patients and had a calculated pooled proportion of 32% percent (95% CI 0.243–0.407; *I*^2^ = 82%; *p* = 0.000) [12, 14, 22, 23, 28, 29, 37, 45, 48, 49, 62, 67, 73, 74, 85, 90, 106]. The last period between 2010 and present included 47 studies with 3747 patients [1, 6, 16, 18–21, 24–27, 31, 35, 36, 42, 46, 47, 50, 52, 53, 55, 57–59, 68, 69, 71, 72, 77, 78, 81–84, 87, 88, 91–95, 97, 98, 100, 103–105]. For this period, the pooled percentage for cardiac anomalies was 34% percent (95% CI 0.290–0.392; *I*^2^ = 89%; *p* = 0.000).

## Discussion

This systematic review with meta-analysis showed an overall pooled percentage of 29% for cardiac anomalies in patients with DO. A persistent foramen ovale was the most frequent diagnosed cardiac anomaly in patients with DO with an occurrence of 35%, followed by atrial septal defect and ventricular septal defects which both occurred in 33% of the patients with DO. DO due to complete atresia (type 3) was the most frequent cause of DO and occurred in 54% of the patients. Trisomy 21 was seen in 28% of the patients with DO. In patients with both DO and trisomy 21, the risk of having a cardiac anomaly of 16%, whereas the pooled percentage of cardiac anomalies without presence of trisomy 21 in these patients was 15%. The most common cardiac anomalies in combination with trisomy 21 were ventricular septal defects and persistent ductus arteriosus with an occurrence of nine percent.

In general population, the incidence of a cardiac anomaly is one per 100 live births and a patent foramen ovale occurs between 25 and 30% [107, 108]. Approximately two-thirds of these children have a mild cardiac anomaly, such as minor ASD or VSD, which is clinically insignificant [109]. Some of these mild anomalies are only discovered later in life, and the actual percentage of congenital cardiac anomalies might be higher. Our findings suggest that patients born with a DO are comparatively more at risk of also having a cardiac anomaly and is found in 29% of the patients. The clinical significance of these cardiac anomalies, however, is uncertain.

According to previous research, 12% of cardiac anomalies are severe leading to hemodynamic challenges or early intervention [110]. ASD and VSD are the two most common cardiac anomalies in the general population which demand further analysis and sometimes surgical intervention. These cardiac anomalies have incidences of two to five cases per 1000 live births, respectively. In our cohort of DO patients, we found a higher occurrence of seven to nine percent for VSD and seven to eight percent for patients with an ASD, depending on the presence of trisomy 21. The combination with DO may have consequences for postnatal management in these children, as hemodynamic insufficiency can cause problems with perioperative anesthesia. Moreover, heart surgery may be required to treat the anomaly. In that case, the DO surgery is postponed, the patient weakened before surgery and might need parenteral nutrition. In the most severe cases, the cardiac anomaly can result in fatal outcome.

In live births, 78% of hemodynamically relevant cardiac anomalies are detected prenatally. This number has increased over the last decades [109]. This detection rate is not known in patients with DO. We found that 29% of these children with DO have cardiac anomalies. This percentage is substantially higher than in the general population, emphasizing the importance of prenatal screening in these children. It is critical that these children are treated in a pediatric surgical center with the presence of pediatric cardiologists.

A recently performed systematic review showed an incidence of cardiac anomalies in patients with trisomy 21 of 60% [111]. This incidence was slightly higher than our calculated pooled percentages of 51% that was found in patients with DO and trisomy 21. One hypothesis for this could be that cardiac anomalies are sometimes asymptomatic, and full screening was not always performed in the past, as we show a pooled percentage of 11% of cardiac anomalies between 1956 and 1979. Due to the retrospective design of most studies, the reported percentages may even be lower than the actual occurrence. A recent performed study in our center supported the finding of equal incidences of cardiac anomalies in patients with DO with and without trisomy 21 [112]. The presence of cardiac anomalies and trisomy 21 combined in this systematic review was only described in 20 studies [10, 11, 17, 19, 21, 23, 29, 34, 38, 39, 49, 64, 66, 67, 71, 76, 79, 85, 91, 93, 102] showing almost equal pooled percentages of 16% for trisomy 21 with cardiac anomalies, trisomy 21 without cardiac anomalies and cardiac anomalies without trisomy 21. This could indicate that the increased occurrence of cardiac anomalies in children with DO is not only associated with trisomy 21, but is also related to DO itself.

A hypothesis about the association between cardiac anomalies and DO is a defect in recanalization of the primitive duodenum, which occurs between the 8th and 10th week of gestation [113]. Studies show intestinal atresia occurs in week six to seven of gestation due to failure of recanalization. [114]. The fact that the failure of recanalization occurs this early in gestation might be an explanation for the fact that DO is highly associated with cardiac anomalies, but also various other anomalies [28]. The development of septal defects ASD and AVSD starts at the fourth week of embryonic life [115]. Based on the early stage of gestation, pathophysiology might exist there. However, relationship between these two in pathogenesis in early gestational age has not yet been proven.

Since the invention of echocardiogram in the 1950s, this medical imaging technique is continuously improving [116]. This might influence the detection rate of cardiac anomalies. We calculated a pooled percentage of 11% in the first time frame between 1956 and 1979, which increased over the years to a pooled percentage of 34% between 2010 and present. In recent years, there have been further advancements in echocardiogram, such as the use of contrast media and introduction of 3D imaging. These significant improvements over time have led to better diagnostic accuracy and increased use of echocardiogram, which might influence the detection rate of cardiac anomalies [116]. However, not all these cardiac anomalies might be hemodynamically or clinically relevant.

The reported pooled proportions based on the available literature bring some limitations—the methodological shortcomings of the majority of the studies, not describing cardiac anomalies in a prospective evaluation, lack of uniformity in the definitions used for classifying DO and cardiac anomalies, and heterogeneity of the studies which undeniably has led to the influence of forms of bias, such as selection, publication, and reporting bias. Our risk of bias assessment showed most articles to have only fair quality. The presented data are the best available approximation of the occurrences of cardiac anomalies in patients with DO.

## Conclusion

This is the first review that investigates the occurrence of cardiac anomalies in patients with DO based on known literature. We show that cardiac anomalies are present in almost one-third of the patients with DO. It is therefore important that preoperative screening for cardiac anomalies in these patients will be part of standard care, regardless of the presence of trisomy 21.

### Supplementary Information

Below is the link to the electronic supplementary material.

Supplementary file1 (DOCX 19 KB)

Supplementary file2 (DOCX 17 KB)

## Data Availability

Data sharing is not applicable to this article as no new data were created or analyzed in this study.
