# Supplementary material for: Cardiac anomalies in children with congenital duodenal obstruction: a systematic review with meta-analysis
Source: Pediatr Surg Int. 2023 Mar 26;39(1):160. doi: 10.1007/s00383-023-05449-3 (PMC10040397; doi:10.1007/s00383-023-05449-3)
Supplement: Supplementary file 1 — Supplementary file1 (DOCX 19 KB) [file 383_2023_5449_MOESM1_ESM.docx]

| **Author** | **Year** | **Design** | **Number DO** | **Number cardiac anomalies** | **Type cardiac anomaly described?** | **Type DO described?** | **Trisomy 21** |
| --- | --- | --- | --- | --- | --- | --- | --- |
| Aitken | 1966 | Retrospective cohort study | 29 | 5 | No | Yes | 6 |
| Akhtar | 1992 | Retrospective cohort study | 49 | 24 | Yes | Yes | 34 |
| Al-Salem | 2007 | Retrospective cohort study | 35 | 7 | No | Yes | 10 |
| Al-Salem | 1989 | Retrospective cohort study | 19 | 4 | No | No | X |
| Arnbjornsson | 2002 | Retrospective cohort study with prospective control | 25 | 7 | Yes | No | 8 |
| Atwell | 1982 | Prospective cohort study | 35 | 3 | No | No | 13 |
| Avci | 2018 | Retrospective cohort study | 32 | 2 | No | Yes | 11 |
| Bailey | 1993 | Retrospective cohort study | 85 | 6 | No | Yes | 15 |
| Bairdain | 2014 | Retrospective cohort study | 87 | 48 | Yes | Yes | 33 |
| Bethell | 2020 | Prospective cohort study | 97 | 47 | Yes | Yes | 33 |
| Bishay | 2013 | Retrospective cohort study | 54 | 28 | No | No | 17 |
| Bishop | 2020 | Retrospective cohort study | 21 | 2 | No | Yes | 6 |
| Bittencourt | 2004 | Retrospective cohort study | 21 | 5 | No | Yes | 7 |
| Brantberg | 2002 | Prospective cohort study | 29 | 9 | Yes | Yes | 6 |
| Burgmeier | 2012 | Retrospective cohort study | 20 | 5 | No | Yes | 7 |
| Burjonrappa | 2011 | Retrospective cohort study | 59 | 29 | No | Yes | 18 |
| Chiarenza | 2017 | Retrospective cohort study | 18 | 1 | No | Yes | 5 |
| Cho | 2017 | Retrospective cohort study | 56 | 8 | No | Yes | X |
| Choudhry | 2009 | Retrospective cohort study | 61 | 19 | No | Yes | 28 |
| Cohen-Overbeek | 2008 | Retrospective cohort study | 91 | 21 | No | Yes | 29 |
| Cragan | 1993 | Retrospective cohort study | 86 | 21 | Yes | Yes | 15 |
| Cresner | 2022 | Retrospective cohort study | 96 | 32 | No | Yes | X |
| Dalla Vecchia | 1998 | Retrospective cohort study | 138 | 53 | No | Yes | 33 |
| Danishmend | 1986 | Retrospective cohort study | 98 | 12 | No | No | 13 |
| Deguchi | 2022 | Retrospective cohort study | 82 | 25 | Yes | Yes | 17 |
| Davey | 1980 | Retrospective cohort study | 68 | 19 | No | Yes | 23 |
| Dewberry | 2020 | Retrospective cohort study | 39 | 11 | No | Yes | 11 |
| Escobar | 2004 | Retrospective cohort study | 169 | 46 | No | Yes | 46 |
| Fogel | 1991 | Retrospective cohort study | 15 | 4 | Yes | Yes | 5 |
| Gavopoulos | 1993 | Retrospective cohort study | 25 | 3 | Yes | Yes | 3 |
| Girvan | 1974 | Retrospective cohort study | 158 | 35 | No | Yes | 50 |
| Grosfeld | 1993 | Retrospective cohort study | 103 | 35 | No | Yes | 31 |
| Hall | 2011 | Retrospective cohort study | 56 | 11 | No | Yes | 13 |
| Hancock | 1989 | Retrospective cohort study | 34 | 8 | No | Yes | 7 |
| Harberg | 1979 | Retrospective cohort study | 39 | 12 | No | Yes | 16 |
| Hemming | 2007 | Retrospective cohort study | 67 | 11 | Yes | Yes | 23 |
| Hill | 2011 | Retrospective cohort study | 58 | 28 | No | Yes | 26 |
| Holler | 2019 | Retrospective cohort study | 44 | 27 | No | Yes | 14 |
| Jimenez | 2004 | Retrospective cohort study | 16 | 4 | No | Yes | 4 |
| Keckler | 2008 | Retrospective cohort study | 94 | 37 | Yes | Yes | 39 |
| Khan | 2017 | Retrospective cohort study | 109 | 40 | Yes | Yes | X |
| Kim | 2016 | Retrospective cohort study | 59 | 28 | Yes | Yes | 9 |
| Kimble | 1997 | Retrospective cohort study | 41 | 16 | Yes | No | 12 |
| Komuro | 2011 | Retrospective cohort study | 57 | 23 | Yes | Yes | 18 |
| Kozlov | 2011 | Prospective cohort study | 27 | 7 | No | No | 10 |
| Kraeger | 1973 | Retrospective cohort study | 19 | 2 | No | Yes | 3 |
| Kumar | 2016 | Retrospective cohort study | 31 | 4 | No | Yes | 6 |
| Kyyronen | 1988 | Retrospective cohort study | 92 | 14 | No | Yes | 17 |
| Lin | 2012 | Retrospective cohort study | 22 | 7 | Yes | Yes | 2 |
| Mahmood | 2021 | Prospective cohort study | 20 | 2 | Yes | No | 5 |
| Makkadafi | 2021 | Retrospective cohort study | 52 | 14 | No | Yes | 21 |
| Merrill | 1976 | Retrospective cohort study | 23 | 5 | No | No | 7 |
| Mikaelsson | 1997 | Retrospective cohort study | 16 | 3 | Yes | No | 7 |
| Miranda | 2008 | Retrospective cohort study | 46 | 15 | No | Yes | 16 |
| Miro | 1988 | Retrospective cohort study | 26 | 3 | No | Yes | 6 |
| Miscia | 2019 | Retrospective cohort study | 140 | 26 | No | Yes | 27 |
| Mooney | 1987 | Retrospective cohort study | 20 | 5 | Yes | Yes | 2 |
| Moore | 1956 | Retrospective cohort study | 32 | 2 | No | Yes | 2 |
| Murshed | 1999 | Retrospective cohort study | 275 | 102 | No | No | 82 |
| Mustafawi | 2008 | Retrospective cohort study | 77 | 51 | Yes | Yes | 36 |
| Muto | 2022 | Retrospective cohort study | 26 | 10 | Yes | No | X |
| Nakamura | 2019 | Retrospective cohort study | 27 | 3 | No | No | 9 |
| Nerwich | 1994 | Retrospective cohort study | 30 | 9 | No | Yes | 6 |
| Niramis | 2010 | Retrospective cohort study | 227 | 94 | No | Yes | 86 |
| Oh | 2017 | Retrospective cohort study | 22 | 11 | No | Yes | 6 |
| Ozturk | 2007 | Retrospective cohort study | 20 | 2 | No | Yes | 5 |
| Piper | 2008 | Retrospective cohort study | 63 | 31 | No | Yes | 22 |
| Rattan | 1995 | Retrospective cohort study | 23 | 4 | No | Yes | 2 |
| Reid | 1973 | Retrospective cohort study | 164 | 36 | No | No | 50 |
| Repucci | 2022 | Retrospective cohort study | 40 | 17 | No | No | X |
| Saalabian | 2022 | Retrospective cohort study | 33 | 21 | No | No | 9 |
| Safra | 1976 | Retrospective cohort study | 10 | 5 | No | No | 3 |
| Samuel | 1997 | Retrospective cohort study | 64 | 10 | No | No | 6 |
| Sarin | 2012 | Retrospective cohort study | 18 | 1 | No | Yes | 2 |
| Savran | 2016 | Retrospective cohort study | 15 | 3 | No | Yes | 4 |
| Short | 2014 | Retrospective cohort study | 67 | 47 | Yes | Yes | 32 |
| Sidler | 2020 | Retrospective cohort study | 41 | 11 | No | No | 11 |
| Singh | 2004 | Retrospective cohort study | 79 | 40 | Yes | Yes | 28 |
| Singleton | 1963 | Retrospective cohort study | 17 | 1 | No | No | 2 |
| Smith | 2019 | Retrospective cohort study | 43 | 16 | No | No | 19 |
| Son | 2017 | Retrospective cohort study | 112 | 6 | No | No | 11 |
| Spigland | 1990 | Retrospective cohort study | 33 | 4 | No | Yes | 7 |
| Spilde | 2008 | Retrospective cohort study | 29 | 7 | No | Yes | X |
| Stephens | 2018 | Retrospective cohort study | 65 | 12 | Yes | No | 20 |
| Takahashi | 2014 | Retrospective cohort study | 31 | 16 | Yes | No | 15 |
| Takahashi | 2010 | Retrospective cohort study | 18 | 6 | No | Yes | 7 |
| Treider | 2021 | Retrospective cohort study | 100 | 35 | No | Yes | 36 |
| Tsai | 2010 | Retrospective cohort study | 30 | 9 | No | Yes | 4 |
| Tulloh | 1994 | Retrospective cohort study | 20 | 6 | Yes | No | X |
| Van der Zee | 2011 | Retrospective cohort study | 28 | 5 | Yes | No | 11 |
| Vinycomb | 2020 | Retrospective cohort study | 110 | 41 | No | Yes | 21 |
| Waever | 1995 | Retrospective cohort study | 67 | 15 | No | Yes | 25 |
| Wang | 2018 | Retrospective cohort study | 152 | 117 | Yes | Yes | 12 |
| Weber | 1986 | Retrospective cohort study | 41 | 4 | Yes | No | 8 |
| Weitzman | 1974 | Retrospective cohort study | 14 | 3 | Yes | Yes | 5 |
| Weller | 2022 | Retrospective cohort study | 267 | 186 | No | No | 79 |
| Williams | 2021 | Retrospective cohort study | 917 | 487 | No | No | X |
| Yigiter | 2010 | Retrospective cohort study | 22 | 12 | No | Yes | 8 |
| Zyromski | 2008 | Retrospective cohort study | 48 | 10 | No | Yes | 10 |
