# Supplementary material for: Cardiac anomalies in children with congenital duodenal obstruction: a systematic review with meta-analysis
Source: Pediatr Surg Int. 2023 Mar 26;39(1):160. doi: 10.1007/s00383-023-05449-3 (PMC10040397; doi:10.1007/s00383-023-05449-3)
Supplement: Supplementary file 2 — Supplementary file2 (DOCX 17 KB) [file 383_2023_5449_MOESM2_ESM.docx]

| **Author** | **Year** | **Selection (0-4)** | **Comparability (0-2)** | **Outcome (0-3)** | **Total (0-9)** |
| --- | --- | --- | --- | --- | --- |
| Aitken | 1966 | ** | - | ** | 4 |
| Akhtar | 1992 | *** | - | ** | 5 |
| Al-Salem | 2007 | *** | - | *** | 6 |
| Al-Salem | 1989 | ** | - | * | 3 |
| Arnbjornsson | 2002 | *** | * | ** | 6 |
| Atwell | 1982 | ** | - | * | 3 |
| Avci | 2018 | ** | - | ** | 4 |
| Bailey | 1993 | ** | - | * | 3 |
| Bairdain | 2014 | *** | * | *** | 7 |
| Bethell | 2020 | **** | * | ** | 8 |
| Bishay | 2013 | *** | - | *** | 6 |
| Bishop | 2020 | **** | - | *** | 7 |
| Bittencourt | 2004 | **** | * | *** | 8 |
| Brantberg | 2002 | ** | - | ** | 4 |
| Burgmeier | 2012 | *** | - | ** | 4 |
| Burjonrappa | 2011 | ** | - | *** | 5 |
| Chiarenza | 2017 | ** | - | ** | 4 |
| Cho | 2017 | ** | - | ** | 4 |
| Choudhry | 2009 | *** | - | ** | 5 |
| Cohen-Overbeek | 2008 | *** | - | ** | 5 |
| Cragan | 1993 | *** | - | * | 4 |
| Cresner | 2022 | *** | - | * | 4 |
| Dalla Vecchia | 1998 | ** | - | ** | 4 |
| Danishmend | 1986 | ** | - | - | 2 |
| Deguchi | 2022 | *** | * | *** | 7 |
| Davey | 1980 | *** | - | - | 3 |
| Dewberry | 2020 | *** | - | * | 4 |
| Escobar | 2004 | ** | - | ** | 4 |
| Fogel | 1991 | *** | * | * | 4 |
| Gavopoulos | 1993 | ** | - | * | 3 |
| Girvan | 1974 | ** | - | - | 2 |
| Grosfeld | 1993 | *** | - | ** | 5 |
| Hall | 2011 | *** | - | * | 4 |
| Hancock | 1989 | ** | - | * | 3 |
| Harberg | 1979 | ** | - | - | 2 |
| Hemming | 2007 | *** | - |  | 4 |
| Hill | 2011 | **** | - | ** | 6 |
| Holler | 2019 | *** | - | * | 4 |
| Jimenez | 2004 | ** | - | * | 3 |
| Keckler | 2008 | *** | - | ** | 5 |
| Khan | 2017 | **** | - | ** | 6 |
| Kim | 2016 | **** | - | *** | 7 |
| Kimble | 1997 | *** | - | * | 4 |
| Komuro | 2011 | *** | - | * | 4 |
| Kozlov | 2011 | **** | ** | ** | 8 |
| Kraeger | 1973 | ** | - | * | 3 |
| Kumar | 2016 | ** | - | * | 3 |
| Kyyronen | 1988 | ** | - | * | 3 |
| Lin | 2012 | *** | - | *** | 6 |
| Mahmood | 2021 | *** | * | ** | 6 |
| Makkadafi | 2021 | *** | - | * | 4 |
| Merrill | 1976 | ** | - | * | 3 |
| Mikaelsson | 1997 | ** | - | ** | 4 |
| Miranda | 2008 | ** | - | * | 3 |
| Miro | 1988 | ** | - | - | 2 |
| Miscia | 2019 | *** | - | ** | 5 |
| Mooney | 1987 | ** | - | - | 2 |
| Moore | 1956 | * | - | * | 2 |
| Murshed | 1999 | *** | - | * | 4 |
| Mustafawi | 2008 | **** | - | *** | 7 |
| Muto | 2022 | **** | - | ** | 6 |
| Nakamura | 2019 | **** | - | *** | 7 |
| Nerwich | 1994 | **** | - | * | 5 |
| Niramis | 2010 | *** | - | ** | 5 |
| Oh | 2017 | *** | - | *** | 6 |
| Ozturk | 2007 | *** | - | ** | 5 |
| Piper | 2008 | *** | - | *** | 6 |
| Rattan | 1995 | ** | - | - | 2 |
| Reid | 1973 | * | - | - | 1 |
| Repucci | 2022 | *** | - | ** | 5 |
| Saalabian | 2022 | *** | - | * | 4 |
| Safra | 1976 | *** | - | * | 4 |
| Samuel | 1997 | *** | - | * | 4 |
| Sarin | 2012 | ** | - | ** | 4 |
| Savran | 2016 | **** | - | *** | 7 |
| Short | 2014 | *** | - | *** | 6 |
| Sidler | 2020 | **** | * | ** | 7 |
| Singh | 2004 | *** | - | *** | 6 |
| Singleton | 1963 | * | - | * | 2 |
| Smith | 2019 | **** | - | *** | 7 |
| Son | 2017 | *** | * | ** | 6 |
| Spigland | 1990 | *** | - | ** | 5 |
| Spilde | 2008 | *** | - | ** | 5 |
| Stephens | 2018 | *** | - | *** | 6 |
| Takahashi | 2014 | *** | - | ** | 5 |
| Takahashi | 2010 | *** | - | ** | 5 |
| Treider | 2021 | *** | - | *** | 6 |
| Tsai | 2010 | **** | - | ** | 6 |
| Tulloh | 1994 | **** | - | ** | 6 |
| Van der Zee | 2011 | ** | - | *** | 5 |
| Vinycomb | 2020 | *** | - | ** | 5 |
| Waever | 1995 | *** | - | ** | 5 |
| Wang | 2018 | ** | - | * | 3 |
| Weber | 1986 | * | - | * | 2 |
| Weitzman | 1974 | ** | - | ** | 4 |
| Weller | 2022 | *** | - | *** | 5 |
| Williams | 2021 | *** | * | *** | 7 |
| Yigiter | 2010 | ** | - | ** | 4 |
| Zyromski | 2008 | *** | - | ** | 5 |
